# Supporting evidence and recommendations for the management of patients with systemic-eligible atopic dermatitis: A Canadian consensus document

**DOI:** 10.1016/j.jdin.2025.06.009

**Published:** 2025-09-26

**Authors:** Kerri Purdy, Melinda Gooderham, Mohannad Abu-Hilal, April Armstrong, Rachel Asiniwasis, Gurbir Dhadwal, Carolyn Jack, Perla Lansang, Fiona Lovegrove, Julien Ringuet, Shanna Spring

**Affiliations:** a Division of Dermatology, Dalhousie University, Halifax, Nova Scotia, Canada; b Department of Dermatology, Queen's University, Kingston, Ontario, Canada; c SKiN Centre for Dermatology and Probity Medical Research, Toronto, Ontario, Canada; d Michael G. DeGroote School of Medicine, McMaster University, Hamilton, Ontario, Canada; e Department of Dermatology, Keck School of Medicine, University of Southern California, Los Angeles, California; f Department of Dermatology, University of Saskatchewan, Regina, Saskatchewan, Canada; g Department of Dermatology and Skin Science, University of British Columbia, Vancouver, British Columbia, Canada; h Division of Dermatology, McGill University, Montreal, Québec, Canada; i Division of Dermatology, Faculty of Medicine, University of Toronto, Toronto, Ontario, Canada; j Lovegrove Dermatology, London, Ontario, Canada; k Centre de Recherche Dermatologique du Québec Métropolitain (CRDQ), Québec City, Québec, Canada; l Department of Pediatrics, Division of Dermatology and Rheumatology, Children's Hospital of Eastern Ontario, University of Ottawa, Ottawa, Canada

**Keywords:** atopic dermatitis, consensus, pathophysiology, practical management, systemic-eligible, systemic therapy, special populations

## Abstract

**Background:**

Atopic dermatitis is a chronic, relapsing, and remitting inflammatory skin disease. Multiple systemic therapeutic options are available to treat atopic dermatitis.

**Objective:**

To provide evidence-based recommendations on the use of systemic therapies for atopic dermatitis in Canada that consider the nuances of the Canadian healthcare system and provide guidance for populations of clinical interest.

**Methods:**

A panel of 14 experts, including 11 dermatologists from Canada and 3 from the United States, reviewed available literature on systemic therapies for atopic dermatitis. The published evidence, along with clinical expertise and opinion, was used to draft a concise set of statements to guide healthcare providers in Canada on systemic treatment of atopic dermatitis.

**Results:**

During 3 rounds of virtual meetings with all 14 experts voting in all meetings, a total of 29 statements reached the 75% agreement required for consensus.

**Limitations:**

The consensus statements are based on expert opinions and consensus in the absence of evidence-based clinical research for patient outcomes. The statements represent considerations for patient management and not specific guidelines for patient treatment.

**Conclusion:**

The recommendations and statements provided serve to guide Canadian healthcare providers on the practical aspects of managing systemic-eligible patients with atopic dermatitis.

**Capsule Summary**

• This article builds on recommendations from international guidelines for the use of systemic therapies for moderate-to-severe atopic dermatitis (AD) and tailors recommendations for a Canadian context.
• These evidence-informed recommendations can support Canadian practitioners in selecting the appropriate systemic treatment for their systemic-eligible patients with AD.

## Introduction

Atopic dermatitis (AD) is a common, chronic, relapsing, and remitting inflammatory skin disease causing pruritus, scaly erythematous lesions, and reduced quality of life in affected patients. The disease can also be associated with atopic comorbidities, such as asthma or allergic rhinitis. In patients with moderate-to-severe AD who do not respond to topical therapy, systemic therapy may be indicated to reduce symptoms, minimize flares, and improve quality of life.^1^ The decision to initiate these systemic therapies relies on evidence-based recommendations, clinical assessments, and risk–benefit profiles, considering specific patient factors and shared decision-making.

Recently, clinical practice guidelines from the American Academy of Allergy, Asthma, and Immunology (AAAAI)^2^ and American Academy of Dermatology (AAD)^3^ were updated to address the use of systemic therapies in AD. However, these guidelines do not address the nuances of the Canadian healthcare system, such as differences in the availability of certain medications, and do not provide complete guidance for populations of clinical interest within Canada.

Here, we provide evidence-based statements derived from a consensus-building exercise of Canadian dermatologists treating systemic-eligible patients with AD, addressing key topics to support management: pathophysiology and treatment selection, systemic options available in Canada, and considerations for key special populations. Rather than being prescriptive, the statements are meant to provide guidance for assessing the risk–benefit profile of systemic therapies to be weighed alongside shared decision-making on an individual patient basis.

## Methods

### Consensus

Literature searches, limited to English-language studies in adult and pediatric populations for systemic management of AD, were performed in February 2024. Professional medical writers independently reviewed the literature and developed consensus statements.

An expert panel on systemic-eligible AD treatment was formed, consisting of 11 Canadian and 3 American dermatologists with clinical and research expertise, who identified topic areas that could benefit from further guidance. Each panelist was assigned 1 of 3 topic areas to review literature and discuss related statements. Statements were anonymously voted on for relevance and validity using a 5-point Likert scale. Consensus agreement was prespecified as 75% indicating relevant or very relevant, and valid or very valid. Statements failing to meet the 75% threshold were revised based on panel comments; any statements that could not be acceptably revised were removed. After the virtual meetings, Canadian experts reviewed all consensus statements across the 3 topics to align on final statements for this article. See Supplementary Appendix, available via Mendeley at https://data.mendeley.com/datasets/7n73mp5ngp/1 for additional details on literature review and consensus generation.

## Consensus statements

### Pathophysiology

**Fig 1:**
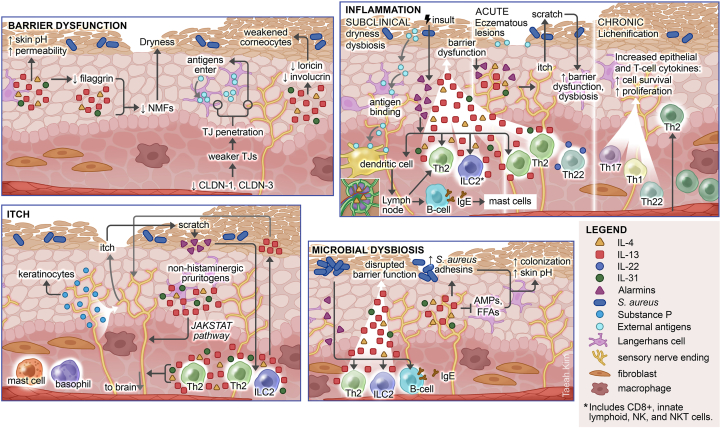
The pathophysiology of atopic dermatitis (AD) involves a complex interplay of barrier dysfunction, inflammation, itch, and microbial dysbiosis.^4^, ^5^, ^6^, ^7^, ^8^, ^9^, ^10^, ^11^ Barrier dysfunction: Both genetic mutations and type 2 cytokines reduce barrier proteins. Filaggrin expression is impaired, lowering natural moisturizing factors (NMFs), increasing skin pH, and trans-epidermal water loss. Decreased loricrin and involucrin impair the strength and flexibility of corneocytes. Serine protease activity and matrix metalloproteins are increased, further reducing cellular adhesion and long-chain ceramide lipids. Tight junctions (TJ) are reduced, increasing permeability; C-fibers and higher numbers of Langerhans cell dendrites penetrate TJs in AD, increasing exposure to environmental antigens and allergen sensitization. Inflammation: Inflammation, microbial dysbiosis, and barrier dysfunction are present at subclinical levels in non-lesional AD skin with xerosis. In response to injury or infection, epidermal keratinocytes produce “alarmin” cytokines and chemokines, activating immune responses. Professional antigen-presenting dendritic cells migrate to the lymph nodes, where IL-4 and OX40 drive Th2 cell differentiation. Type 2 cytokines drive pathology, impairing barrier functions, and promoting *S. aureus* expansion, which triggers more injury and alarmins. The IL-13–rich milieu in AD skin triggers keratinocytes to downregulate proteins such as filaggrin and activates fibroblasts in the dermis. Alarmins and cytokines from activated cells recruit additional effectors from the circulation, increasing Th1, Th17, and Th22 cytokines, driving cellular proliferation and epidermal thickening, inflammation, and the cycle of neuro-immune itch hypersensitivity. Itch: Various itch pathways exist in AD, but histamine-independent pruritogens predominate and sensitize or activate C-fibers, including IL-31, type 2 cytokines, and alarmins, leading to severe itch and scratching behavior. IL-31 has direct pruritogenic effects (modulated by JAK1/2-STAT). In addition to inflammatory itch signaling, neuropeptides produced by C-fibers contribute to activating immune cells such as mast cells, driving cutaneous inflammation. Microbial dysbiosis: *S. aureus* can damage skin via a range of toxins and proteases and lipases and produces superantigen that drives polyclonal inflammation and IgE-sensitization. Type 2 cytokines promote *S. aureus* adhesins and inhibit epidermal antimicrobial peptides and free fatty acids, further increasing skin pH and susceptibility to *S. aureus* colonization. *NK*, Natural killer.

AD pathophysiology is heterogeneous and multifactorial, involving epidermal barrier dysfunction, immune function dysregulation, disrupted microbial composition, genetic predisposition, and environmental factors (Fig 1, Table I).^16^^,^^17^

**Table I:** Type 2 cytokines involved in atopic dermatitis pathogenesis

Cytokine	Source	Signaling Pathway	Target	Effect
IL-4	• Mainly produced by TH2-type lymphocytes, type 2 innate-lymphoid cells, and T follicular helper cells^7^ • To a lesser extent by eosinophils, basophils, mast cells, natural killer cells, and CD8+ T lymphocytes^7^	• Type 1: IL-4Rα chain/common gamma chain (γc) subunit (IL-2Rγ), linked to JAK1/3-STAT6 pathway^6^^,^^12^ • Type 2: IL-13Rα1 and IL-4Rα, linked to JAK1/2/TYK2-STAT6^6^^,^^12^	• Hematopoietic cells • Eosinophils • Mast cells • B cells • Sensory Neurons • Smooth muscle • Epithelial cells • Th cells^7^^,^^12^	• Directly activate cutaneous sensory nerves, initiating itch–scratch cycle that enhances barrier dysfunction^7^ • Contribute to the skin recruitment of eosinophils^7^ • T-cell differentiation to Th2 and survival^12^^,^^13^ • Class switching to IgE^13^ • B-cell proliferation^12^ • Eosinophilia • Mast cell proliferation, activation, recruitment, and survival • Basophil activation and recruitment • Downregulation of filaggrin expression^7^
IL-13	• Th2 cells • Basophils • Eosinophils • Mast cells • Epithelial cells^7^	• Type 2: IL-13Rα1 and IL-4Rα, linked to JAK1/2/TYK2-STAT6^6^^,^^12^	• Hematopoietic cells • Th cells • B cells • Eosinophils • Epithelial cells^7^^,^^12^	• Eosinophilia • IgE production • Downregulation of filaggrin expression • Barrier dysfunction • Reduction of lipid production • Itch^7^
IL-31	• Produced by Th2 cells and mature dendritic cells^12^ • Basophils • Eosinophils • Mast cells^7^ • Keratinocytes	• IL-31Ra/OSMR-B: JAK1/2-STAT1/3/5^6^^,^^7^	• Epidermal keratinocytes • Dorsal root ganglia • Basophils • Eosinophils • Mast cells^7^	• Tissue remodeling^14^ • Epidermal barrier function^14^ • Decreasing filaggrin expression^15^ • Itch^15^ • Hyperkeratosis

*Ig*, Immunoglobulin; *IL*, interleukin; *JAK*, Janus kinase; *OSMR*, oncostatin-M receptor; *STAT*, signal transducer and activator of transcription; *Th*, T-helper; *TYK*, tyrosine kinase.

Improved knowledge of immune and inflammatory pathways involved in AD pathogenesis has supported the development of cytokine-targeted systemic therapies. However, due to the complexity of the disease and target diversity, no single optimal treatment has yet been developed. The statements in Table II summarize evidence informing the use of systemic therapies in systemic-eligible patients with AD, including immunosuppressants and advanced targeted agents.

**Table II:** Statements and supporting evidence on the pathophysiology of atopic dermatitis and respective mechanisms of action for advanced targeted systemic agents

Statement	Supporting evidence
1. AD develops through an interplay of genetic factors, inflammation, barrier dysfunction, microbial dysbiosis, and itch. 2. The epithelium is globally defective in AD, leading to increased permeability of environmental agents (e.g., irritants, allergens, microbes), contributing to dysfunctional inflammatory responses which, in turn, exacerbate barrier dysfunction.	• Mutations in certain genes such as *FLG*, *FLG2*, *CLDN1*, and *SPINK5* can lead to disruptions in the skin barrier and allow for environmental insults to penetrate the skin more easily.^18^^,^^19^ • Mutations in genes involved in innate and adaptive immunity, including IL-4, IL-13, IL-31, and TSLP, may also contribute to the pathogenesis of AD through dysregulation of these signal transduction pathways.^18^^,^^20^, ^21^, ^22^, ^23^, ^24^ • Environmental toxins, including cigarette smoke, pollutants, allergens, and microbial agents penetrating the skin barrier can lead to a Th2 immune response, a primary response underlying the development of AD.^25^
3. The type 2 inflammatory response is characterized by aberrant production of cytokines including, but not limited to, IL-4, IL-13, and IL-31. In AD and other type 2 inflammatory diseases, IL-4 and IL-13 signaling is a key initiating pathway.	• Skin barrier disruption during acute disease phases stimulate keratinocytes to release alarmins such as TSLP, IL-33, and IL-25, activating innate-lymphoid and myeloid cells.^26^^,^^27^ • These cells recruit Th2 cells from the blood, which produce the key cytokines IL-4, IL-13, and IL-31.^28^^,^^29^ • Both IL-4 and IL-13 signal via intracellular JAK-STAT pathways primarily involving JAK1, specifically, the heterodimeric receptors IL-4Rα/γc and IL-4Rα/IL-13Rα1.^6^^,^^30^
4. Type 2 cytokines contribute to structural and functional barrier dysfunction through decreasing expression of key epidermal proteins and recruiting immune cells that cause disruption of tight junctions, highlighting the interrelated effects of the immune system and barrier dysfunction in AD.	• The epithelial response to type 2 cytokines leads to the downregulation of barrier proteins such as FLG, loricrin, and involucrin, and promotes production of short-chain fatty acids.^4^ • In areas of active AD, type 2 inflammation leads to the recruitment of eosinophils and mast cells, which release mediators that downregulate stratum corneum structural proteins, disrupt TJs in the epidermis, and further activate and recruit immune cells.^4^
5. IL-4 and IL-13 also act directly on sensory neurons, increasing their sensitivity to several pruritogens (including IL-31) and contributing to the perpetuation of chronic itch in AD. Scratching induced by chronic itch can worsen barrier dysfunction, promote *S. aureus* colonization, and further enhance type 2 inflammation.	• Itch, a major symptom for patients with AD, leads to physical damage to the skin barrier and nidus for potential secondary infections. • Pruritogenic sensory neurons have receptors for several inflammatory mediators released in AD, including IL-4, IL-13, IL-31, IL-33, and TSLP.^4^ • IL-4 and IL-13 can also sensitize neurons, making them more responsive to itch-inducing substances (e.g., IL-31, histamine).^4^ • Disruption of the skin barrier allows for penetration of external foreign substances (e.g., allergens, irritants) into the epidermis, leading to an increased induction of type 2 inflammatory responses, creating a positive feedback loop.^4^
6. The precise role of *S. aureus* in AD remains unclear, but there is a strong association between *S. aureus* and disease severity. *S. aureus* colonization can lead to barrier dysfunction and promote type 2 inflammation, while type 2 inflammation can increase susceptibility to *S. aureus* colonization.	• *S. aureus* skin colonization is observed in up to 20% of the general population, but in up to 90% of patients with moderate-to-severe AD,^31^, ^32^, ^33^, ^34^, ^35^ and correlates strongly with AD severity, biomarkers of type 2 immunity (i.e., eosinophil count, CCL17, periostin, TARC, eotaxin-3), skin barrier disruption, and allergen sensitization.^4^ • *S. aureus* can directly activate itch and damage the epidermis through the release of toxins, proteases, lipases, and superantigens, increasing the epidermal expression of alarmins and cytokines and thus further disrupting skin barrier function and worsening AD.^4^
7. This immune response is not limited to visibly affected skin—the systemic immune dysregulation in AD results in subclinical inflammation in non-lesional skin. Additionally, the immune response may contribute to progressive development of chronic inflammatory comorbidities such as asthma, allergic rhinitis, and food allergy. 8. Early disease control may help reduce impairments associated with the persistent, life-long burden of AD. The perceptions that children outgrow AD can lead to undertreatment, possibly resulting in inadequately controlled disease throughout the patient’s developmental years. There is evolving evidence that suggests minimizing systemic inflammation can disrupt cutaneous susceptibility to type 2 inflammation, which may halt the “atopic march.”	• There is growing evidence that supports the concept of AD as a systemic disease.^36^ • AD is often accompanied by other Th2–driven processes that affect other organ systems as the first step of the atopic march, the progression of allergic diseases from infancy into childhood. The disrupted skin barrier, inflammation, itch, and dysbiosis in AD facilitate allergen sensitization and development of other atopic diseases, such as asthma, food allergy, and allergic rhinitis.^37^ • Evidence on the impact of early systemic intervention to halt the atopic march is still limited and inconsistent, focusing on reduction of known comorbidities like asthma with dupilumab.^38^ • Inflammation in AD is not limited to type 2 processes and may also involve other pathways, including Th1, Th17, and Th22.^36^^,^^39^^,^^40^ Given the systemic nature of inflammation in AD, patients’ overall health should be considered if they develop signs and symptoms of comorbidity.^36^
9. Dupilumab is a monoclonal antibody that inhibits IL-4 and IL-13 signaling by specifically binding the IL-4Rα subunit shared by the IL-4 and IL-13 receptor complexes. 10. Tralokinumab and lebrikizumab are monoclonal antibodies that specifically bind to IL-13 and block downstream effects. Tralokinumab prevents IL-13 from binding to IL-13Rα1 (type 1 receptor) and IL-13Rα2 (decoy receptor), while lebrikizumab prevents heterodimerization of IL-4Rα/IL-13α1. 11. Abrocitinib and upadacitinib are highly selective JAK inhibitors with greater inhibitor potency at JAK1 relative to JAK2, JAK3, and TYK2.	• Systemic treatments targeting the pathways described above, including monoclonal antibodies targeting IL-4 and/or IL-13 and oral JAK1 inhibitors, are effective at improving the signs and symptoms of AD (including itch), as well as patient quality of life, including mental health.^41^, ^42^, ^43^, ^44^, ^45^ • These findings reinforce the important roles of these targets in the pathophysiology of AD and the power of systemic therapies in reducing patients’ overall AD burden.

*AD*, Atopic dermatitis; *CCL*, C-C motif chemokine ligand; *FLG*, filaggrin; *IL*, interleukin; *JAK*, Janus kinase; *STAT*, signal transducer and activator of transcription; *TARC*, thymus and activation-regulated chemokine; *Th*, T-helper; *TJs*, tight junctions; *TSLP*, thymic stromal lymphopoietin.

## Treatment options

A range of effective options for AD management are provided by traditional immunosuppressive medications and newer systemic options targeting the type 2 signaling pathways. Patients with moderate-to-severe AD—as defined by lesional severity, impact on quality of life (QoL), and location of active disease—inadequately controlled with, or unsuitable for, topical prescription therapies may consider systemic options to control their symptoms and improve QoL.^3^^,^^46^ The panel defined these patients as “systemic-eligible.”

In Canada, reimbursement for prescription medications is a patchwork of public and private insurance programs. Public reimbursement is recommended federally; provinces and private insurers typically follow those recommendations. Reimbursement for dupilumab is recommended only after failing previous topical or immunosuppressant therapies,^47^ while neither lebrikizumab nor tralokinumab is publicly reimbursed.^48^^,^^49^ Patients are required to start or transition to the lowest-cost option (typically a biosimilar).^50^ Physicians managing patients with systemic-eligible AD must consider access to the medication in terms of cost to the patient and eligibility for insurance coverage, including progressing patients rapidly through topical treatments to meet prior therapy requirements.^47^^,^^50^

**Fig 2:**
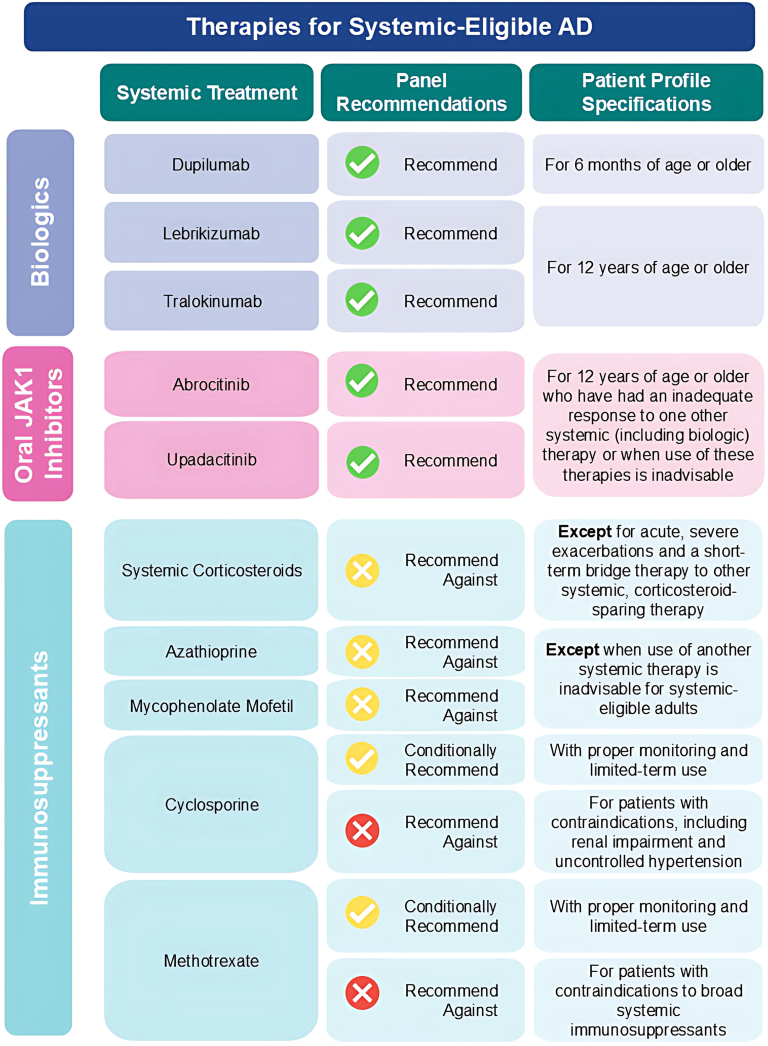
Panel recommendations on the use of therapies for systemic-eligible atopic dermatitis available in Canada. The expert panel reviewed and made recommendations on the use of therapies for systemic-eligible atopic dermatitis available in Canada, including the use of immunosuppressants, oral JAK1 inhibitors, and biologics. Panel recommendations included “Recommend,” Recommend Against,” and “Conditionally Recommend” for patient-profile specifications. *AD*, Atopic dermatitis, *JAK*, Janus kinase.

Statements in Table III and summarized in Fig 2 describe the panel’s recommendations on the use of monoclonal antibodies and oral Janus kinase (JAK)1-selective inhibitors approved in Canada for AD, as well as conventional systemic therapies commonly used off-label for AD, for systemic-eligible patients.

**Table III:** Statements and treatment considerations based on the panel’s recommendations on the use of systemic agents in atopic dermatitis

Statement	Considerations for use
12. We recommend dupilumab for systemic-eligible patients with AD 6 mo of age or older.	• The first Health Canada-approved targeted systemic treatment for AD for adults, adolescents, and children, including infants older than 6 mo of age.^1^^,^^41^^,^^51^ • Ocular surface disease, including conjunctivitis and keratitis, was more frequent with dupilumab versus placebo, but is typically mild to moderate and not warranting treatment discontinuation. Incidence tends to be lower in younger age groups.^1^^,^^51^, ^52^, ^53^, ^54^, ^55^, ^56^, ^57^, ^58^ • Dupilumab is effective in patients with systemic-eligible AD and type 2 comorbidities (asthma, allergic rhinosinusitis with nasal polyposis, eosinophilic esophagitis, and prurigo nodularis),^41^ and is the preferred option for eligible patients with these conditions. • Given the strength of the clinical data and relatively low risk of harm, the panel recommends dupilumab for systemic-eligible patients age 6 mo and older.
13. We recommend anti–IL-13 biologics (tralokinumab and lebrikizumab) for systemic-eligible patients with AD 12 y of age or older.	• Tralokinumab and lebrikizumab are approved in Canada for AD in adults and adolescents age 12 y and older.^42^^,^^45^ • Similar to dupilumab, ocular surface disease is 1 of the few emergent safety concerns.^59^, ^60^, ^61^, ^62^ • Given the strength of the clinical data and relatively low risk of harm, the panel recommends these therapies for systemic-eligible patients age 12 y and older.
14. We recommend oral JAK1-selective inhibitors (abrocitinib and upadacitinib) for systemic-eligible patients with AD 12 y of age or older who have had an inadequate response to 1 other systemic (including biologic) therapy or when use of these therapies is inadvisable.	• Abrocitinib and upadacitinib are approved in Canada for AD in adults and adolescents age 12 y and older who have had an inadequate response to other systemic drugs, including biologics.^43^^,^^44^ • Prophylactic herpes zoster vaccination is recommended before initiating these therapies.^43^^,^^44^ • Based on malignancy and cardiovascular warnings in the product monographs, caution may be warranted in some patients.^3^^,^^43^^,^^44^ • Laboratory monitoring of complete blood count with differential, liver enzymes (upadacitinib only), and lipids are recommended.^43^^,^^44^ • Further guidance on JAK inhibitors in patients with a history of cancer or infection is described in statements 21-24. • Abrocitinib and upadacitinib may be associated with teratogenic risk and must not be used during pregnancy (see statement 28). • Given the indication and strength of the clinical data, the panel recommends these therapies in systemic-eligible patients who have had an inadequate response to another systemic (including a biologic), with recommended monitoring.
15. We recommend against systemic corticosteroids for systemic-eligible patients with AD except for acute, severe exacerbations and a short-term bridge therapy to other systemic, corticosteroid-sparing therapy.	• Given the risk of serious AEs and scarce data demonstrating efficacy, the panel recommends against their use outside of exceptional circumstances, aligned to expert opinion from the International Eczema Council.^63^
16. We recommend against the use of mycophenolate mofetil (MMF) and azathioprine (AZA) for patients with AD except when use of another systemic therapy is inadvisable for systemic-eligible adults.	• Require ongoing laboratory monitoring due to elevated risk of cancer, serious infections, and cytopenias.^3^ • Given the smaller benefit associated with MMF and AZA compared with biologics and JAK inhibitors and their risk of serious AEs, the panel recommends against their use unless other systemic therapy is inadvisable.
17. We conditionally recommend cyclosporine with proper monitoring and limited-term use (less than 1 year of continuous dosing) for systemic-eligible adults with AD. We recommend against use of cyclosporine in patients with contraindications, including renal impairment and uncontrolled hypertension.	• Requires baseline and ongoing laboratory and blood pressure monitoring due to risk of renal impairment.^64^ • Guidelines recommend limiting treatment to no longer than 12 mo.^2^^,^^3^^,^^65^ • Given cyclosporine’s efficacy based on limited data, the panel conditionally recommends its use with appropriate patients and monitoring, for a maximum of 12 mo.
18. We conditionally recommend methotrexate (MTX) with proper monitoring for systemic-eligible adults with AD. We recommend against use of MTX in patients with contraindications to broad systemic immunosuppressants.	• Baseline and ongoing laboratory monitoring is required due to elevated risk of serious infections, cytopenias, and hepatic damage.^2^^,^^3^^,^^65^ • MTX is teratogenic and must not be used during pregnancy, with further guidance described in statement 28. • Given methotrexate’s modest efficacy and potentially more favourable AE profile than other immunosuppressants, the panel conditionally recommends its use in appropriate patients with monitoring.

*AD*, Atopic dermatitis; *AEs*, adverse events; *IL*, interleukin; *JAK*, Janus kinase.

### Monoclonal antibodies

Across age groups, dupilumab showed large improvements in AD signs, symptoms, and QoL compared with placebo in large, double-blind, randomized controlled trials (RCTs) in patients with moderate-to-severe AD inadequately controlled by topical medication(s).^1^^,^^51^ Long-term efficacy has been demonstrated in clinical trials over 5 years for adults and 1 year for adolescents, children, and infants.^52^^,^^53^^,^^56^^,^^57^ Dupilumab was shown to be well tolerated, with an acceptable safety profile and few emergent safety concerns.^1^^,^^51^, ^52^, ^53^, ^54^, ^55^, ^56^, ^57^, ^58^ In a systematic review and network meta-analysis (NMA), dupilumab showed slightly greater efficacy over tralokinumab but lower than that for high-dose JAK inhibitors.^2^^,^^65^

In adults and adolescents, tralokinumab and lebrikizumab showed improvements in AD signs, symptoms, and QoL versus placebo in large, double-blind RCTs in patients with moderate-to-severe AD inadequately controlled by topical medication(s).^60^, ^61^, ^62^ Long-term efficacy in adults has been demonstrated up to 6 years for tralokinumab and up to 2 years for lebrikizumab in clinical trials.^66^^,^^67^ Similar to dupilumab, these biologics are well tolerated.^59^, ^60^, ^61^, ^62^ In an NMA, lebrikizumab showed slightly greater efficacy over tralokinumab, similar efficacy to that of dupilumab, and lower efficacy than that of high-dose JAK inhibitors.^68^

### Oral JAK1-selective inhibitors

In adults and adolescents, abrocitinib and upadacitinib showed rapid improvements in AD signs, symptoms, and QoL compared with placebo in large, double-blind RCTs of patients with moderate-to-severe AD inadequately controlled by topical medication or who had received systemic therapies.^69^, ^70^, ^71^, ^72^, ^73^ Long-term efficacy in clinical trials has been demonstrated over 2 years for both agents, and safety up to 4 and 5 years has been shown for abrocitinib and upadacitinib, respectively.^74^, ^75^, ^76^, ^77^, ^78^

Abrocitinib and upadacitinib include warnings for serious infections, malignancies, thrombosis, and major adverse cardiovascular events (MACE). Both agents showed an increased risk of serious and opportunistic infections, mostly eczema herpeticum. Malignancy and cardiovascular warnings are based on tofacitinib-associated events in an older rheumatoid arthritis (RA) cohort.^3^^,^^43^^,^^44^ Baricitinib, an oral JAK1/2 inhibitor, is approved in Europe for the treatment of moderate-to-severe AD in patients age 6 months and older and is approved and available in Canada for RA and alopecia areata but not for AD.^79^^,^^80^

Based on head-to-head randomized studies and a large systematic review and NMA, high-dose upadacitinib and abrocitinib showed the highest efficacy among systemic agents at reducing AD signs and symptoms but also demonstrated higher rates of adverse events (AEs) compared with biologic treatments.^2^^,^^65^^,^^70^^,^^81^

### Immunosuppressants and antimetabolites

Clinical trial evidence for the use of systemic corticosteroids for AD is limited.^3^^,^^82^ In a systematic review and NMA, improvements in AD severity were seen with little to no improvement for QoL, itch, or sleep disturbance.^2^^,^^65^ This therapy is associated with an elevated risk of serious AEs, including sepsis, venous thromboembolism, gastric ulcer, and fracture, even at relatively low doses.^83^^,^^84^

Mycophenolate mofetil (MMF) and azathioprine (AZA) are occasionally used as off-label treatments for AD with limited evidence. In small randomized, controlled studies, MMF was shown to be as effective as cyclosporine as maintenance therapy, while AZA improved AD severity, itch, and QoL compared with placebo.^85^^,^^86^ Modest improvement in AD severity for both therapies—lower than that for biologics and JAK inhibitors—was reported in a systematic review and NMA. No data on itch or QoL were analyzed for MMF.^2^^,^^65^^,^^87^ Both agents may be associated with elevated cancer risk and can increase the risk of serious infections and cytopenias, among other side effects such as renal and hepatic effects.^2^^,^^3^

Cyclosporine is commonly used as an off-label treatment for AD with limited trial evidence; small randomized studies demonstrate efficacy over methotrexate (MTX) at Week 8 but equal efficacy at later time points, superiority over systemic prednisone, and similar efficacy to MMF.^82^^,^^87^Another study demonstrated improvements in disease biomarkers.^88^ A systematic review and NMA reported that high-dose cyclosporine may be among the more effective systemic options across multiple outcomes, while also rating it as 1 of the most harmful compared with other systemic treatments.^2^^,^^65^ Cyclosporine is associated with an elevated risk of renal impairment, which can increase with cumulative dose, and hypertension.^64^

MTX use for AD also has limited clinical trial evidence, including 2 small, randomized studies showing similar efficacy to cyclosporine at Week 20 but not at Week 8, as well as similar efficacy to AZA.^89^^,^^90^ Modest improvements across various outcomes were reported in a systematic review and NMA.^2^^,^^65^ While serious AEs are uncommon, use of MTX is associated with an elevated risk of serious infections, cytopenias, and hepatic damage, among others.^91^

### Special populations

**Fig 3:**
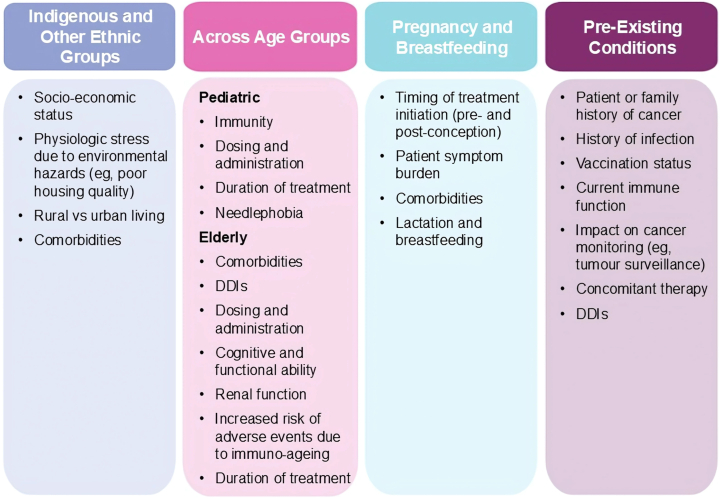
Panel considerations to assist practitioners in treatment selection in special populations in atopic dermatitis. The expert panel provided considerations for using systemic therapies to treat atopic dermatitis in special populations, including indigenous and other ethnic groups, age groups, pregnancy and breastfeeding, and pre-existing conditions. *DDI*, Drug-drug interaction.

The panel identified several Canadian populations where AD treatment modifications should be considered. These populations include indigenous individuals (with elevated socioeconomic and geographic challenges)^92^; patients with skin-of-color (SoC); patients with certain infectious diseases; as well as children, older adults, breastfeeding, or pregnant patients.^93^ Statements in Table IV describe the panel’s recommendations for the management of these populations with systemic therapies. Fig 3 summarizes some key considerations from the panel to assist patients in systemic treatment selection, given certain populations.

**Table IV:** Statements and considerations for systemic treatment based on the panel’s recommendations in various special populations in atopic dermatitis

Statement	Considerations for systemic treatment
19. Many indigenous patients with AD in Canada face a significant burden due to environmental factors, complex social and historical determinants, and poor healthcare and specialist access. Given the high rates of moderate-to-severe disease and secondary skin infections reported in rural and remote communities, prompt and effective treatment is needed for these patients. Therapeutic selection should incorporate culturally competent decision-making.	• Early systemic AD treatments that prioritize safety, manage flares, and reduce the risk of skin infections should be considered in eligible cases.^93^, ^94^, ^95^ • Indigenous and remote communities may have less access to basic treatments for AD or those treatments may be practically non-viable.^94^
20. In North America, patients with skin of color (SoC) (ie, those on the Fitzpatrick scale III-VI, representing darker skin tones) with AD face a significant burden due to various environmental and socioeconomic factors. Given the high rates of lack of representation in educational resources, poor disease control, persistence, delayed diagnosis, and risk of post-inflammatory dyspigmentation, prompt and effective treatment is needed for these patients. Therapeutic selection should incorporate culturally competent decision-making.	• The impact of AD on patients with SoC may be more profound compared to other patients, and warrant prompt and effective systemic treatment for eligible individuals. • To provide equitable access to appropriate treatments, dermatologists should take care to ensure clinician-reported outcomes, such as erythema scoring, are measured appropriately in these patients.
21. For patients with systemic-eligible AD and previous or existing cancer (excluding non-melanoma skin cancer), biologics targeting IL-4/13 are the preferred option based on mechanism of action and limited data available. Caution should be taken when using immunosuppressive systemic therapies that affect interferon pathways and NK cells, as these factors are important in tumor surveillance.	• The panel suggests that biologic treatments offer the lowest additional risks associated with malignancy when treating AD with systemic agents.
22. For patients at risk for or with untreated latent TB, biologic therapies are the preferred option based on their mechanism of action. 23. For patients with HBV infection who are surface antigen positive, concomitant antiviral therapy should be evaluated before starting any oral systemic treatment for AD. Consult with an infectious disease specialist before initiating any systemic therapy. 24. There is an increased risk of HZ for patients receiving JAK inhibitors, but not biologics. HZ vaccination in eligible adults is recommended before starting systemic therapy with JAK inhibitors. Consider prophylaxis for patients with a history of frequent or severe HSV.	• MTX, cyclosporine, and AZA should be avoided in patients with TB history, particularly when alternative biologic agents are available.^3^^,^^96^ • Anti-TB should be considered at least 1 month before initiation of a JAK inhibitor for a patient with latent TB.^43^^,^^44^^,^^97^ • In patients with chronic or resolved HBV infection, concomitant HBV treatment should be considered before initiating systemic treatment for AD.^97^^,^^98^ • Use of biologics targeting Th2 responses (e.g., dupilumab, tralokinumab, and lebrikizumab) are warranted for HBV.^97^ • HZ infection rates are lower in patients treated with dupilumab compared to placebo,^93^^,^^99^ while JAK inhibitors have been found to result in higher rates of HZ infections.^70^^,^^73^^,^^81^ • Physicians should screen for HSV history and consider prophylaxis or other antiviral treatment, especially with prior episodes of eczema herpeticum.^97^
25. For older adults (65 y of age and older) with systemic-eligible AD, biologic therapies are the preferred option based on their safety profile, with no concerns regarding drug interactions or comorbidities, such as renal or hepatic dysfunction. The long-term risk of broad immunosuppression is unknown in these patients. 26. For infants and children (age 6 mo to 11 y) with systemic-eligible AD and after inadequate response to topical treatment, dupilumab is currently the preferred and only approved option. Due to the lack of long-term outcomes data in children, broad-acting immunosuppressants should only be considered following inadequate response to all other available options. 27. For adolescents (age 12 to 17 y) with systemic-eligible AD and after inadequate response to topical treatment, biologics and JAK inhibitors are the preferred options (JAK inhibitors are recommended for patients who have had an inadequate response to a biologic or when use of a biologic is inadvisable). Traditional immunosuppressants should only be considered following inadequate response to biologics or JAK inhibitors due to the risk of side effects in these patients.	• Of the systemic AD treatments available, dupilumab has the most data supporting use in elderly patients. Other agents, including MTX and MMF, may be used at lower doses or with increased monitoring. AZA and cyclosporine should not be used due to toxicity.^97^ • Although JAK inhibitors are indicated for use in older populations, there is a higher risk of AEs, so care should be taken for these patients. • Systemic therapy is warranted in pediatric patients with inadequate disease control; at the time of writing, dupilumab is the only Health Canada-approved systemic treatment for AD in infants and children older than 6 mo of age.^41^ • Most agents, including MTX, cyclosporine, AZA, and MMF are not indicated for treatment of pediatric AD, but have been used off-label.
28. For pregnant patients with systemic-eligible AD, biologics are the preferred systemic option, as no evidence of harm has been shown during pregnancy. Based on their pharmacologic properties and safety profile, biologics are preferred over traditional immunosuppressants. JAK inhibitors, MTX, and MMF are contraindicated in pregnancy.	• Dupilumab appears to be safe during pregnancy, as no major adverse outcomes with pregnant patients or their newborns have been reported.^100^ • Cyclosporine has most frequently been used for the treatment of AD in pregnancy as there has been no evidence of mutagenesis or teratogenic effects and no impacts on nursing.^97^ • Prednisolone is acceptable for treatment of flares during pregnancy.^101^ • MTX, MMF, and JAK inhibitors must not be used during pregnancy. For women using these agents who are considering pregnancy, washout periods of 1 month (JAK inhibitors) or 3 mo (MTX and MMF) prior to conception are recommended.^101^
29. For breastfeeding patients with systemic-eligible AD, biologics are the preferred systemic option as no evidence of harm has been shown during breastfeeding. Based on their pharmacologic properties and safety profile, biologics are preferred over small molecules such as JAK inhibitors and traditional immunosuppressants. JAK inhibitors are contraindicated in breastfeeding.	• During breastfeeding, cyclosporine and dupilumab are appropriate choices, with some evidence to support AZA as an option as well.^101^

*AD*, Atopic dermatitis; *AEs*, adverse events; *AZA*, azathioprine; *HBV*, hepatitis B virus; *HSV*, herpes simplex virus; *HZ*, herpes zoster; *IL*, Interleukin; *JAK*, Janus kinase; *MMF*, mycophenolate mofetil; *MTX,* methotrexate; *NK*, natural killer; *SoC*, skin of color; *TB*, tuberculosis; *Th*, T-helper.

### Indigenous and remote communities

Indigenous populations represent 5% of the Canadian population but are the fastest-growing segment.^102^ AD is a significant concern in rural and remote indigenous communities, as patients experience a higher risk of secondary skin infections due to limited healthcare access, crowded housing conditions, and inadequate access to clean water.^94^^,^^95^

Indigenous peoples often experience health disparities due to complex healthcare policies and challenges rooted in historical, social, and legal factors.^103^^,^^104^ Many indigenous populations reside in rural or remote areas,^105^ requiring long-distance travel for medical care, leading to less frequent follow-ups.^103^ Hyperinflated shipping costs make access to many basic skin care products, such as moisturizers or barrier creams, challenging in remote regions.^94^ Furthermore, though the Canada Non-insured Health Benefits (NIHB) provides insurance coverage of pharmaceuticals for First Nations and Inuit peoples, there are restrictions in the coverage of medications, including no coverage for tralokinumab or lebrikizumab, and limited coverage for dupilumab (limited to individuals over the age of 12 years despite Health Canada indication for age of 6months or older).^106^

### Skin-of-color

Skin-of-color (SoC) is an important consideration for AD treatment due to the increasing diversity of the Canadian patient population. Patients with SoC represent over 25% of the Canadian population (Table V). AD exhibits different morphologic presentations across races and skin tones,^109^ resulting in challenges with recognizing erythema.^110^ There is a higher prevalence and persistence of severity of AD among SoC populations, particularly Black populations, East Asians, and Southeast Asians.^18^^,^^19^^,^^40^^,^^111^^,^^112^ Individuals adapted to warmer and more humid climates may have more difficulty adjusting to the cold and dry climates of Canada, exacerbating the production of inflammatory mediators and contributing to conditions like AD.^113^ Socioeconomic status and stigmatization due to more visible dyspigmentation and xerosis may contribute to disparities in access to healthcare.^111^^,^^114^ Many clinicians are increasingly addressing AD in skin tones where they may have limited experience and may not recognize the severity or impact of the disease on the individual patient.

**Table V:** Prevalence of skin-of-color in the Canadian population^107^^,^^108^

Racial group	Approximate representation in Canada	Approximate percent of Canadian population
South Asian	2.6 million	7%
Middle Eastern	1.7 million	5%
Chinese	1.7 million	5%
Black	1.5 million	5%
Southeast Asian	1.3 million	4%

### Malignancy

There are limited data in the AD population on treatment-associated cancer risks.^97^^,^^115^, ^116^, ^117^, ^118^ Malignancy should be considered when determining the treatment course for AD. Immunosuppressive AD treatments have intrinsic risk in patients with a history of malignancy. However, the risk of malignancy is more strongly associated with patient characteristics, such as age or smoking history.^119^ Current biologic treatments are not immunosuppressive and are associated with lower malignancy risk.^120^ Retrospective dupilumab studies and case report series demonstrate no increased risks in treating patients with prior or active malignancy or in long-term clinical trials.^56^^,^^96^^,^^120^^,^^121^ While an association between interleukin (IL)-4 blockade and cutaneous T-cell lymphoma (CTCL) has been suggested, a large retrospective analysis and other literature suggest this may be due to an “unmasking” effect of patients with CTCL initially misdiagnosed with AD and failing therapy; skin biopsy should be used in equivocal cases for accurate diagnosis.^122^

### Infectious diseases

Patients with active herpes zoster (HZ), history of or active hepatitis B virus (HBV), active or frequent/severe herpes simplex virus (HSV), or tuberculosis (TB) should be treated with caution, particularly when considering traditional systemic treatments, which may exacerbate these infections.^97^ TB reactivation is a concern for MTX, cyclosporine, and AZA, which exhibit broad immunosuppressive activity.^123^ Reactivation of latent HBV in those with chronic or resolved infection may be a concern with certain systemic therapies affecting T-helper (Th)1 pathways, so biologics targeting Th2 responses may be preferred.^97^

Untreated AD is associated with an increased risk of HZ infection.^97^ Head-to-head studies have shown increased frequency of HZ with JAK inhibitors and not with dupilumab.^70^^,^^81^ Immune responses to HSV may be modified by systemic AD treatment, though serious HSV infections were rare with biologics and JAK inhibitors.^97^

*S. aureus* skin colonization is observed in most patients with moderate-to-severe AD. Bacterial infections caused by *S. aureus* are another common comorbidity, causing impetigo, cellulitis, or skin abscesses.^124^^,^^125^ Systemic therapies may be effective at reducing colonization in patients with AD, with 1 RCT illustrating a rapid reduction in *S. aureus* with dupilumab, correlating with reductions in biomarkers and disease severity.^125^

### Geriatric and pediatric AD

Elderly and pediatric populations account for approximately 40% of the total Canadian population.^126^ Statements 25 to 27 recommend factors that should be considered in the management of AD in children, adolescents, and older adults, which should focus on using agents with adequate efficacy and safety data. In elderly patients, age-related changes such as cognitive decline, cancer history, immunosenescence, comorbidities, and polypharmacy, as well as the ability to adhere to a medication, should be considered.^97^

Dupilumab has been shown to significantly improve AD in children as young as 6 months, with an acceptable safety profile,^54^ while currently, all biologics and JAK inhibitors approved for AD in Canada have demonstrated efficacy in patients 12 years of age and older.^58^^,^^60^^,^^69^^,^^71^^,^^72^^,^^127^

### Pregnancy and breastfeeding

Assessment of safety of AD treatments in pregnancy is difficult due to limited evidence.^97^ A systematic review including 68 patients with 69 pregnancies showed dupilumab was not associated with major adverse outcomes in these patients or in their newborns, though studies with extended follow-up were recommended.^100^ Other biologic agents would be expected to have similar outcomes, but no data are available to confirm this.^97^ Systemic treatment using MTX, MMF, and JAK inhibitors is contraindicated, and washouts of up to several months are recommended before conception. Statements 28 and 29 make recommendations for the treatment of patients with AD who are pregnant, considering pregnancy, or currently breastfeeding.

## Conclusion

In Canada, nuances such as access to primary healthcare, healthcare funding mechanisms, and ethnic diversity are not completely reflected in international guidelines. In this article, we present guiding statements for physicians to reflect on their current management of patients with moderate-to-severe AD who are eligible for systemic therapy and identify the appropriate treatment for any given individual. These statements are not prescriptive but can serve as a framework for the assessment of individual patients to make informed and rational treatment decisions.

## Conflicts of interest

Dr Purdy has been an advisor and/or speaker for AbbVie, Lilly, Sanofi Genzyme, and Pfizer. They have also served as principal investigator and received research grant support for AbbVie and Sanofi Genzyme. Dr Gooderham has received grant support from AbbVie, Acelyrin, Amgen, AnaptysBio, Arcutis, Aristea, ASLAN, Bausch Health, Boehringer Ingelheim, Bristol- Myers Squibb, Cara Therapeutics, Dermavant Sciences, Inc., Lilly, Galderma, LP, Incyte, Inmagene Biopharmaceuticals, Janssen, LEO Pharma, Meiji Seika Pharma Co., Ltd, MoonLake Immunotherapeutics, Nimbus Therapeutics, Novartis, Pfizer, Regeneron, Reistone Biopharma, Sun Pharma, Takeda, Tarsus, UCB, Ventyx Biosciences, and VYNE Therapeutics, and has received consulting fees from Bausch Health, Boehringer Ingelheim, Bristol Myers Squibb, Janssen, Novartis, and Sanofi Genzyme. They have also served as speaker and/or advisor for AbbVie, Amgen, Arcutis Biotherapeutics, Boehringer Ingelheim, Bristol Myers Squibb, Lilly, Galderma Laboratories, LP, Incyte, Janssen, LEO Pharma, Novartis, Pfizer, Regeneron, Sanofi Genzyme, Sun Pharma, Takeda, and UCB. Dr. Abu-Hilal has been an advisor and/or speaker for Sanofi. Dr Armstrong has served as a consultant and/or speaker for AbbVie, Amgen, Arcutis, Bristol Myers Squibb, Dermavant, Lilly, Galderma, Janssen, LEO Pharma, Novartis, Pfizer, Regeneron, Sanofi, and UCB. They have served as principal investigator and received research grant support from Bristol Myers Squibb, Janssen, Novartis, and Takeda, and have served as data safety monitoring board member for Boehringer Ingelheim and Parexel. Dr Asiniwasis has been an advisor and/or speaker for Sanofi, Lilly, Pfizer, and AbbVie, and has been a principal investigator for Sanofi and Pfizer. Dr Dhadwal has served as consultant for AbbVie, Pfizer, Sanofi, Lilly, and LEO Pharma. Dr Jack has received grants from Fonds de Recherche du Québec–Santé (FRQS), SkIN Canada, Programme d’aide à l’entrepreneuriat (PAEN), Mackenzie I. Watson Educational Grant, Innovaderm Research, McGill University Department of Medicine, McGill CPD MedUpdates, MITACS, Canadian Dermatology Foundation, Canadian Dermatology Association, and Eczema Society of Canada, and has been involved in clinical studies and/or consultancy work for Sanofi, Lilly, AbbVie, Novartis, Valeant, Bausch, Pfizer, Amgen, Celgene, Janssen, Boehringer Ingelheim, Asana, LEO Pharma, Dermavant, AntibioTx, Neokera, Kiniksa, Ralexar, Arcutis, BMS, Boston, Cara, Concert, Incyte, Sienna, Aristea, Target PharmaSolution, Lyceum Health, La Roche-Posay, Johnson & Johnson Inc., L'Oréal, Chronicle, Catalytic, Beiersdorf, Innomar, Apogee Therapeutics, Galderma, LEAD, and UCB. Dr. Lansang has received honoraria and/or consulting fees from AbbVie, Amgen, Arcutis, Bausch Health, Boehringer Ingelheim, Bristol Myers Squibb, Celgene (Amgen), Lilly, Galderma, Incyte, Janssen, LEO Pharma, Novartis, Pfizer, Regeneron, Sandoz, Sanofi Genzyme, Sun Pharma, and UCB. Dr Lovegrove has been an advisor and/or speaker for AbbVie, Arcutis, Bausch Health, Incyte, LEO Pharma, Lilly, Pfizer, and Sanofi, and has served as consultant and/or investigator for AbbVie, Incyte, LEO Pharma, Lilly, and Sanofi. Dr Ringuet has been a speaker, advisor, and/or principal investigator for AbbVie, Amgen, Arcutis, Apogee, Alumis, Aristea, ASLAN, Bausch Health, Boehringer Ingelheim, Bristol Myers Squibb, Celgene, CONCERT, Correvitas, DICE Tx, Lilly, Galderma, Incyte, Innovaderm, Janssen, JAMP, LEO Pharma, Kyowa Kirin, Merck, Novartis, Pfizer, Sanofi, Sun Pharma, and UCB, and has received research grant support from AbbVie, Amgen, Apogee, Alumis, and Aristea. Dr Spring has served as an advisor for AbbVie, Pfizer, Sanofi, and Lilly, and as a speaker for Sanofi.
